# Headspace Solid-Phase Micro-Extraction Versus Hydrodistillation of Volatile Compounds from Leaves of Cultivated *Mentha* Taxa: Markers of Safe Chemotypes

**DOI:** 10.3390/molecules27196561

**Published:** 2022-10-04

**Authors:** Adam Kowalczyk, Piotr Kuś, Zvonimir Marijanović, Carlo I. G. Tuberoso, Izabela Fecka, Igor Jerković

**Affiliations:** 1 Department of Pharmacognosy and Herbal Medicines, Faculty of Pharmacy, Wroclaw Medical University, 50-556 Wroclaw, Poland; 2 Department of Food Technology and Biotechnology, Faculty of Chemistry and Technology, University of Split, Ruđera Boškovića 35, 21000 Split, Croatia; 3 Department of Life and Environmental Sciences, University of Cagliari, University Campus, S.P. Monserrato-Sestu Km 0.700, 09042 Monserrato, CA, Italy; 4 Department of Organic Chemistry, Faculty of Chemistry and Technology, University of Split, Ruđera Boškovića 35, 21000 Split, Croatia

**Keywords:** *Mentha* spp., essential oil, HS-SPME, GC-MS, PCA

## Abstract

Various mint taxa are widely cultivated and are used not only for medicinal purposes but also in cosmetic and industrial applications. The development of new varieties or cultivars of mint generates difficulties in their correct identification and safe use. Volatile organic compounds (VOCs) from the leaves of seven different taxa of the genus *Mentha* obtained by hydrodistillation (HD) and headspace solid-phase microextraction (HS-SPME) were analyzed using gas chromatography–mass spectrometry (GC-MS). Principal component analysis (PCA) was also performed. Comparative GC-MS analysis of the obtained extracts showed similarity in the major compounds. PCA data allowed the separation of two groups of chemotypes among the analyzed mints, characterized by the abundance of piperitenone oxide and carvone. Two out of seven analyzed taxa were not previously examined for VOC profile, one was examined only for patent application purposes, and six out of seven were investigated for the first time using the HS-SPME technique. The presented analysis provides new data on the abundance and qualitative characterization of VOCs in the studied mint plants and on the safety of their use, related to the possibility of the presence of potentially toxic components. HS-SPME is a valuable method to extend the characterization of the VOC profile obtained by hydrodistillation.

## 1. Introduction

Main volatile organic compounds (VOCs), synthesized by various aromatic plants, are used in many areas of everyday life, including medicine, food, and cosmetics. They can be found in many forms, from freshly harvested or dried plants to extracts and herbal preparations, which may include essential oils (Eos). VOCs are complex mixtures of mainly terpenes, such as mono- and sesquiterpenes and phenylpropane derivatives [1]. Monoterpenes are one of the groups commonly found in Eos. These compounds can be classified as hydrocarbons and oxygen derivatives such as alcohols, phenols, aldehydes, ketones, acids, or terpene oxides. They are responsible for the aroma, flavor, and biological properties of the plants and the preparations derived from them, e.g., essential oils [2,3,4]. Some of these properties can be beneficial therapeutically (expectorant, antiseptic, diuretic, digestive stimulants, etc.), but some may have toxic effects [5].

The next most abundant compounds present in plant volatile fractions, in addition to monoterpenes, are sesquiterpenes. They exhibit a wide range of biological activities, such as anti-inflammatory, antioxidant, antibacterial, antifungal, antiviral, and antineoplastic, and they broaden the potential uses of plants that contain them, including those from the genus *Mentha* [6]. Most terpenes are classified as safe natural chemicals. Some of the biological properties of monoterpenes and sesquiterpenes, such as antifungal and antimicrobial, have been shown to be the result of their disruption of the cell membrane or cytoplasmic structures as a consequence of oxidative stress, while others can assist radical scavengers and are also known as antioxidant molecules [2,7].

Taxa of the genus *Mentha* are among the most frequently cultivated and used aromatic plants, but their systematics is quite complex. In particular, new cultivars created mainly for food or cosmetic purposes are difficult to correctly identify and to evaluate for their safety. They are mainly developed to obtain specific organoleptic properties, such as odor or taste. Thus, the composition of their VOCs is not always known, and it is not clear whether the compounds they contain are safe for the consumers’ health. Mint leaves are well known for the presence of VOCs characterized by a wide variety of monoterpenes and sesquiterpenes, as well as polyphenolic compounds including flavonoids, e.g., eriocitrin, luteolin and their glycosides, and phenolic acids such as rosmarinic acid [8,9].

Various methods are used to extract VOCs from plant materials such as hydrodistillation (HD), supercritical fluid extraction (SFE), and solid-phase micro-extraction (SPME) techniques. HD is a well-known and widely used process to obtain the essential oils from aromatic plants for both laboratory and industrial purposes. It has the advantage of relatively low cost and no chemical contamination of the extract. However, some volatile compounds may be lost at high extraction temperatures or are temperature-sensitive and can be degraded, which has a significant impact on the quality of the essential oil obtained in this way [10]. SPME has been routinely used for the analysis of VOCs in headspace (HS) analysis in laboratory studies and does not require organic solvents. The main differences between the HS-SPME procedure and hydrodistillation are the abundance and qualitative variations of extracted components from plant materials. These depend on the process conditions such as SPME fiber, extraction temperature, and time [11].

The aim of this study was to identify the most suitable method to investigate VOCs in mint leaves and to provide qualitative and abundance data on the major compounds, as well as potentially toxic ones. The analyses were carried out by comparing the volatile profile from leaves of seven different cultivated *Mentha* taxa, obtained using two methods, headspace solid-phase microextraction (HS-SPME) and hydrodistillation (HD), followed by gas chromatography–mass spectrometry analysis (GS-MS). Two out of seven analyzed taxa were not examined previously for VOCs profile (*M. × piperita* var. *officinalis* f. *pallescens* Camus ‘Swiss’, *M. × carinthiaca*), one was examined only for patent application purposes (*M. dulcia citreus* Hillary’s Sweet Lemon), and six out of seven were investigated for the first time using HS-SPME. The statistics included the evaluation of the collected data using principal component analysis (PCA).

## 2. Results and Discussion

### 2.1. Essential Oil Content

**Figure 1:**
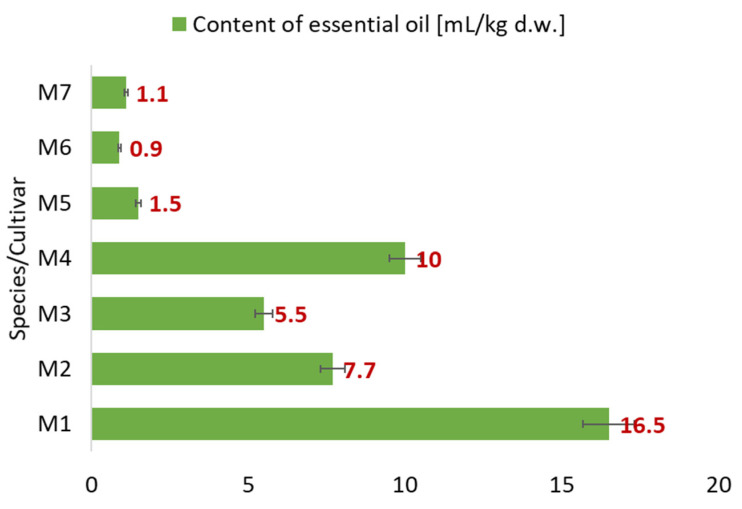
Essential oil content in analyzed mint samples obtained by HD method: **M1** *Mentha* × *piperita* var. *officinalis* f. *pallescens* Camus ‘Swiss’, **M2** *Mentha* × *piperita* f. *citrata* ‘Grapefruit’, **M3** *Mentha* × *piperita* f. *citrata*, **M4** *Mentha* × *carinthiaca* Host., **M5** *Mentha dulcia citreus* Hillary’s Sweet Lemon, **M6** *Mentha longifolia* var. *schimperi* Briq., and **M7** *Mentha longifolia* L.; d.w., dried weight; *n* = 3.

Figure 1 presents the quantitative content of the EOs in the analyzed samples determined using the HD method and expressed in mL/kg of dry plant matter. The highest essential oil content was observed for *M. × piperita* var. *officinalis* f. *pallescens* Camus ‘Swiss’ (**M1**) (16.5 mL/kg) and the lowest for *M. longifolia* var. *schimperi* (**M6**) (0.9 mL/kg). In *M. × carinthiaca* (**M4**), essential oil content was 10 mL/kg, and, in the other species, it ranged from 1.1 mL/kg in *M. longifolia* (**M7**) to 7.7 mL/kg in *M. × piperita* f. *citrata* ‘Grapefruit’ (**M2**).

### 2.2. GC-MS Analysis of the Volatile Compounds Obtained Using HD and HS-SPME

The EOs obtained using the HD method were subjected to quantitative and qualitative analysis via GC-MS and the results are presented in Table 1.

Menthol is generally recognized as safe (GRAS), and its toxic effects on humans have rarely been reported [12,13]. Among the analyzed Eos, only the oil from *M. × piperita* var. *officinalis* f. *pallescens* Camus ‘Swiss’ (**M1**) was characterized by *p*-menthone as the main compound (41%) and by menthol (28.19%).

Another monoterpene often found in taxa of the genus *Mentha* is linalool, widely applied in the cosmetic, food, and pharmaceutical industries. In an animal toxicology study, it was shown to cause up to a fourfold increase in serum aspartate aminotransferase enzyme levels and a twofold decrease in body weight in animals when fed with 5% linalool. An increase in this enzyme can lead to myocardial necrosis, liver damage, skeletal muscle damage, or pancreatitis [7]. Linalool (40.43%) was the dominant constituent of the EO from *M. × piperita* f. *citrata* (**M3**), which is consistent with the literature [14].

Carvone can be found quite commonly in the EOs from different mints and has been shown to have multidirectional biological and therapeutic effects including antibacterial, antifungal, antioxidant, anti-inflammatory, and antihypertensive [15]. In *M. × piperita* f. *citrata* ‘Grapefruit’ (**M2**) and in *M. × carinthiaca* (**M4**), carvone predominated: 62.92% and 72.13%, respectively. *cis*-Dihydrocarvone (26.87%) and dihydrocarvyl acetate (13.01%) were found to be the main components in *M. dulcia citreus* Hillary’s Sweet Lemon (**M5**). Carvone as the major component was identified in *M. × piperita* var. *officinalis* f. *pallescens* Camus (63.31%) (**M1**) and in *M.× piperita* f. *citrata* (**M3**) (77.61%). Jakowienko’s research also revealed carvone to be the predominant compound in a similar taxon, but HD was performed for the whole herb (stems and leaves) after collection of plant material before flowering [16]. Moreover, Mogosan et al. reported that, in this taxon, carvone (41.21%) and menthol (12.77%) are the compounds found in the highest amounts [17]. *cis*-Dihydrocarvone was also the predominant component determined in *M. dulcia citreus* Hillary’s Sweet Lemon (**M5**) by Westerfield [18].

Pulegone and menthofuran, which may have hepatotoxic effects, are found not only in *M. pulegium* but also in smaller amounts in other taxa of the genus *Mentha* [19]. The European Medicines Agency, in a public statement on the use of herbal medicinal products containing pulegone and menthofuran, described the toxicity of this compounds and recommended limit values for herbal medicinal products that contain them [20]. According to the toxicological conclusions of the EMA’s statement, the target organs for pulegone and menthofuran are the liver and kidney. Several chemotypes of *M. longifolia* can be distinguished including the piperitenone-dominated chemotype [21,22]. Piperitenone (38.92%), piperitenone oxide (32.88%) and *cis*-piperitone oxide (21.05%) were present in the highest amounts in *M. longifolia* var. *schimperi* (**M6**), while, in the EO from *M. longifolia* (**M7**), only piperitenone oxide was detected as the predominant compound (84.66%). Other studies identified pulegone as the major EO compound from this taxon, as well as isomenthone or carvone [23,24,25,26]. Menthofuran was not detected in any analyzed Eos obtained by HD, while pulegone was detected only in *M. × piperita* var. *officinalis* f. *pallescens* Camus ‘Swiss’ (**M1**) (0.62%). In this taxon, pulegone (2.14%) was also identified by Mogosan et al. [17]. The EO from the leaves of *M. longifolia* was characterized by pulegone as the main component by Sayed et al. [27].

Thymol, 1,8-cineole (syn. eucalyptol), and limonene are very often used in the food and cosmetic industries because of their characteristic organoleptic properties. However, they also show various pharmacological properties: mucolytic, antibacterial, antiviral, antifungal, and anti-biofilm [28,29,30]. Thymol is one of the main constituents of the essential oils of the genus *Thymus*; however, it can also be found in small amounts in the genus *Mentha* [31,32,33,34]. In all taxa except *M. longifolia* var. *schimperi* (**M6**), 1,8 cineole was present. Limonene was detected in *M. × piperita* var. *citrata* ‘Grapefruit’ (**M2**) at the level 9.11%, *M. dulcia citreus* Hillary’s Sweet Lemon (**M5**) at 6.89%, *M. × carinthiaca* (**M4**) at 5.64%, *M.* × *piperita* var. *citrata* (**M3**) at 1%, and *M. longifolia* var. *schimperi* (**M6**) at 0.34%. The highest amount of thymol in analyzed samples was present in *M. × piperita* var. *citrata* (**M3**), at 7.04%.

The results of the GC-MS analysis of the volatile fractions obtained using HS-SPME are presented in Table 2. HS-SPME parameters were optimized with respect to the overall number of identified compounds. The standard deviation achieved was <10% for triplicate measurements. To the best of our knowledge, VOCs of taxa **M1–M5** and **M7** were investigated for the first time using the HS-SPME technique. Cordero’s study showed that piperitenone oxide was found to be predominant among VOCs of *M. longifolia* (**M7**) leaves, which was also confirmed by the performed analysis [35]. Najafian et al. identified pulegone as the major component of VOCs obtained using the HS-SPME method from this taxon [23]. Comparing GC-MS results of mints VOCs obtained using HD and HS-SPME, it was found that their main components were the same, and they differed only in their abundance. Some smaller variations were observed in the profile of the constituents. Menthofuran (7.27%) and a higher amount of pulegone (3.67%) compared with HD were observed only in *M. × piperita* var. *officinalis* f. *pallescens* Camus ‘Swiss’ (**M1**). The concentrations of α-pinene, sabinene, β-pinene, and β-myrcene were lower in the samples from the mint *M. × piperita* var. *officinalis* f. *pallescens* Camus ‘Swiss’ (**M1**), *M. × piperita* var. *citrata* ‘Grapefruit’ (**M2**), *M. × piperita* var. *citrata* (**M3**), *M. × carinthiaca* (**M4**), and *M. dulcia citreus* Hillary’s Sweet Lemon (**M5**). Limonene was not determined in *M. longifolia* (**M7**). In all VOCs obtained using HS-SPME, 1,8-cineole was present, but in lower amounts compared to the HD, except for *M. longifolia* var. *schimperi* (**M6**), where this compound was determined only using HS-SPME. Similar amounts of (*Z*)-β- and (*E*)-β-ocymenes were observed for each taxon. Higher *cis*-piperitone oxide abundance was detected using HS-SPME for *M. longifolia* (**M7**). In *M. longifolia* var. *schimperi* (**M6**) and *M. longifolia* (**M7**), β-bourbonene was absent. In all analyzed taxa, *trans*-caryophyllene was detected. Germacrane D was not present in *M. longifolia* var. *schimperi* (**M6**).

**Figure 2:**
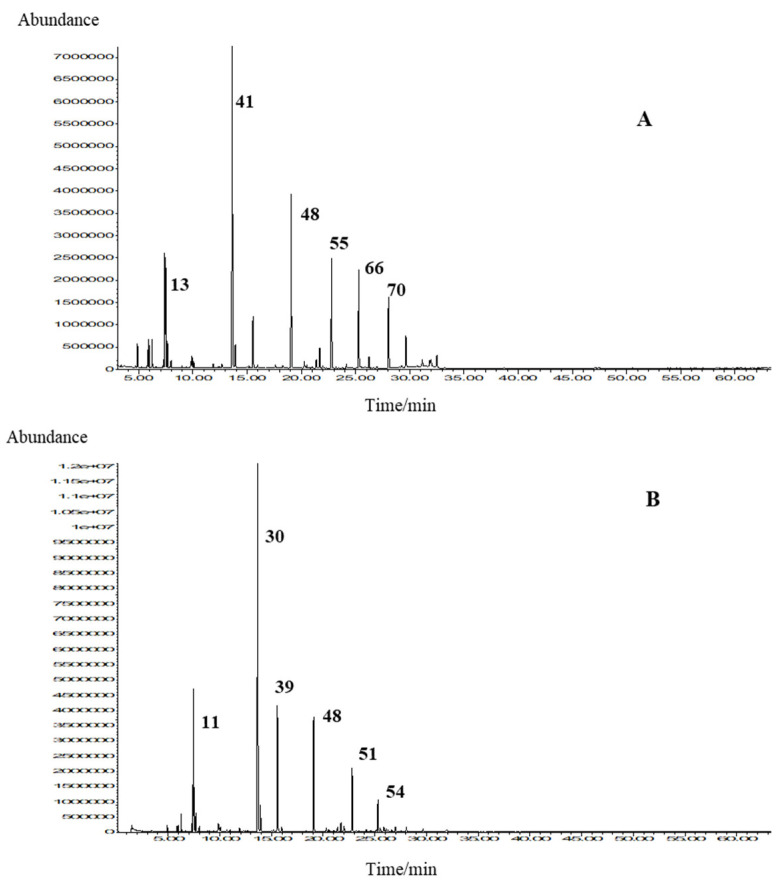
Total ion chromatograms (TICs) of the headspace (**A**) and essential oil (**B**) of *Mentha dulcia citreus* Hillary’s Sweet Lemon (**M5**). The numbers in (**A**) correspond to the compounds in Table 2, and the numbers on (**B**) correspond to Table 1.

Selected chromatograms of the sample **M5** are presented in Figure 2.

### 2.3. Principal Component Analysis

**Figure 3:**
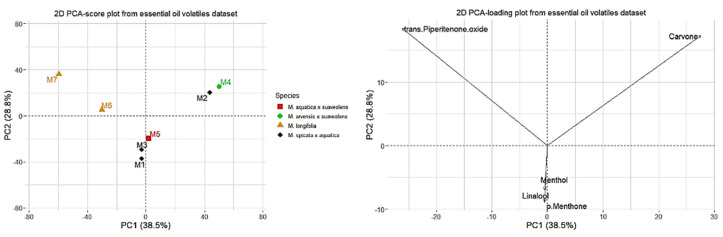
Principal component analysis (PCA) scores (**left**) and loadings (**right**) (only first five variables with the highest contributions are shown) plots based on essential oil volatiles dataset.

**Figure 4:**
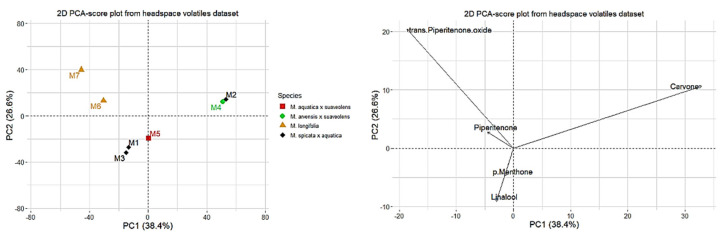
Principal component analysis (PCA) scores (**left**) and loadings (**right**) (only first five variables with the highest contributions are shown) plots based on headspace volatiles dataset.

The datasets containing volatile constituents determined via GC-MS analyses of the fractions obtained using hydrodistillation (EO) and HS-SPME were subjected to PCA. The data in both cases demonstrated some natural clustering into three groups (Figure 3 and Figure 4). In the case of the data obtained for the EO, the first two PCA factors explained 67. 3% of variance between the samples and the data obtained for the headspace extracts; the main two factors explained 65.0% of variance in the dataset. The variables providing the greatest contribution to the first two principal components were the following for EO: carvone (accounting for more than 45% and 35% of components 1 and 2, respectively) and piperitenone oxide (accounting for more than 50% and more than 40% of factors 1 and 2, respectively), along with *p*-menthone, linalool, and menthol (accounting in total for about 20% of factor 2). In the case of HS-SPME data, the variables providing the greatest contribution to the first two principal components were carvone (accounting for more than 70% and 15% of components 1 and 2, respectively) and piperitenone oxide (accounting for more than 20% of component 1 and nearly 60% of component 2), along with linalool, accounting for more than 10%, and *p*-methone, *cis*-dihydrocarvone, and menthol, accounting for less than 5% each of component 2. In Figure 3 and Figure 4, the first five compounds exhibiting major contribution to the variance are presented. The samples naturally formed three groups: chemotypes differentiated by the abundance of piperitenone oxide, carvone or linalool, and menthol, *p*-menthone, and other related compounds. The clustering was similar based on both EO and HS-SPME data. The first group characterized by the abundance of piperitenone oxide contained *M. longifolia* taxa (*M. longifolia* var. *schimperi* **M6**, *M. longifolia* **M7**). The second group consisted of *M.* × *piperita* f. *citrata* ‘Grapefruit’ (**M2**) and *M.* × *carinthiaca* (**M4**), which are hybrids of *M. spicata* and *M. aquatica* or *M. arvensis* and *M. suaveolens*, respectively. The last group consisted of *M.* × *piperita* var. *officinalis* f. *pallescens* Camus ‘Swiss’ (**M1**), *M.* × *piperita* f. *citrata* (**M3**) two *M. piperita* (*M. spicata* × *aquatica*) cultivars, and *M. dulcia citreus* Hillary’s Sweet Lemon **M5** (*M. aquatica* × *suaveolens*) [36].

## 3. Materials and Methods

### 3.1. Plant Material

The leaf samples of seven cultivated mints were obtained from the collections at the Botanical Garden of Medicinal Plants (Wrocław, Poland), Wroclaw Medical University (M. Stachura, Wrocław, Poland), Botanical Garden, Wroclaw University (J. Kochanowska, Wrocław, Poland) and Ogrody Ziolowe in Wrocław (M. Dewódzki, Wrocław, Poland), Poland in late July or early August during flowering in 2021. The following taxa were used for the analysis: *Mentha* × *piperita* var. *officinalis* f. *pallescens* Camus ‘Swiss’ (**M1**), *Mentha* × *piperita* f. *citrata* ‘Grapefruit’ (**M2**), *Mentha* × *piperita* f. *citrata* (**M3**), *Mentha* × *carinthiaca* Host. (**M4**), *Mentha dulcia citreus* Hillary’s Sweet Lemon (**M5**), *Mentha longifolia* var. *schimperi* Briq. (**M6**), and *Mentha longifolia* L. (**M7**). The mint leaves were previously dried in the shade, in air at room temperature of 22 ± 2 °C and a humidity of 40–45%. The voucher specimens were placed in the Department of Pharmacognosy and Herbal Medicines, Wroclaw Medical University, Poland.

### 3.2. Essential Oil Hydrodistillation (HD)

From dried leaves of seven taxa of the genus *Mentha*, EOs were obtained using a modified pharmacopeial hydrodistillation method according to the monograph of *Menthae piperitae folium* in the European Pharmacopoeia [37]. Briefly, 20.0 g of dried and crushed plant material was added to a 500 mL flask with 200 mL of water. It was connected to a Deryng apparatus with a water cooler and heated in a laboratory electric heating mantle with thermal controller at the boiling point of the water. Distillation was carried out for 2 h. No xylene was used, but pentane and diethyl ether (1:2, *v*/*v*) were trapped in a stack. The results obtained from three replicates are presented as mean values.

### 3.3. Headspace Solid-Phase Microextraction (HS-SPME)

An automated PAL RSI SPME holder (CTC Analytics AG, Zwingen, Switzerland) was used containing DVB/CAR/PDMS fiber (Divinylbenzene/Carboxen/Polydimethylsiloxane, 50/30 μm, Supelco Co., Bellefonte, PA, USA). Each sample of the leaves was cut in small pieces, and ~2 g was placed in a 20 mL clear screw top vial and hermetically sealed with PTFE/silicone septum. The automated PAL RSI was programmed as follows: conditioning SPME fiber (according to Supelco Co. Instructions, Bellefonte, PA, USA); sample agitation (agitation speed, 250 rpm; agitator on time, 5 s; agitator off time, 2 s; equilibration, 30 min at 40 °C; VOC extraction, 40 min without agitation; injection of the fiber in the GC injector for desorption, 7 min. The results are presented as mean values of three replicates.

### 3.4. GC-MS Analysis

An Agilent Technologies (Palo Alto, CA, USA) gas chromatograph model 7820A equipped with a mass selective detector (MSD) model 5977E (Agilent Technologies, Palo Alto, CA, USA) was used. An HP-5MS capillary column (Agilent J & W GC column, Palo Alto, CA, USA; 5%-phenyl-methylpolysiloxane; 30 m × 0.25 mm, 0.25 μm film thickness) was used for GC-MS analysis. The GC conditions were as follows: split ratio, 1:50; oven programmed, 2 min at 70 °C, with the temperature increased at the rate of 3 °C/min to 200 °C and held isothermal for 15 min; injector temperature, 250 °C; detector temperature, 300 °C; carrier gas, He (velocity: 1 mL/min). The MSD (EI mode) was operated at 70 eV, and the mass range was 30–300 average mass units (amu). Firstly, 1 μL of diluted essential oil (10 μL of the oil in 1 mL of pentane) was manually inserted with a syringe into the GC injector. The identification of the VOCs was based on the comparison of their retention indices (RIs) determined relative to n-alkanes (C9–C25) with those reported in the literature and their mass spectra were determined via comparison with the spectra from Wiley 9 (Wiley, New York, NY, USA) and NIST 17 (D-Gaithersburg) mass spectral libraries. The percentage composition of the samples was computed from the GC peak areas using the normalization method (without correction factors). The average component percentages were calculated from three GC-MS analyses.

### 3.5. Statistics

The data were mean-centered and evaluated using PCA. The analyses were performed using R for Windows, version 4.0.0 (R-Cran project, http://cran.r-project.org/ accessed on 3 October 2022) including the “factoextra” library [38].

## 4. Conclusions

GC-MS analysis of VOCs from selected taxa of the genus *Mentha* obtained using two methods—headspace solid-phase micro-extraction (HS-SPME) and hydrodistillation (HD)—showed differences in both abundance and profile of the determined compounds. The predominant group among the analyzed terpenes comprised monoterpenes) linalool, *p*-menthone, menthol, *cis*-dihydrocarvone, carvone, *cis*-piperitone oxide, dihydrocarvyl acetate, piperitenone, and piperitenone oxide_. The main components in the volatile fractions obtained using the two methods—HS-SPME and HD—were the same, but their abundances were different. The leaves from two taxa—*M.* × *piperita* var. *officinalis* f. *pallescens* Camus ‘Swiss’, *M.* × *carinthiaca*—were studied for their volatile compound profile for the first time, and the VOC profiles of HS-SPME extracts from six of the seven taxa were not comparable with those obtained using HD. On the one hand, the HD technique is considered a time-consuming and laborious process. It also requires a larger sample of plant material and is not suitable for thermolabile compounds. On the other hand, HS-SPME is a faster technique that does not require large amounts of material and is more protective for analyzed compounds. The combination of these two techniques provides a more complete profile of plant VOCs. The performed analysis demonstrated that more components were determined using HS-SPME than HD; therefore, both techniques can supplement the analysis of VOCs in plant material. In terms of potentially toxic volatile compounds, all taxa were found to be safe.

## Figures and Tables

**Table 1:** Volatile component composition (peak area%) obtained using the HD method and their amount in *Mentha* spp.

No.	Compound	RI		Sample Symbol and Content (%)						
			RI_L_	M1	M2	M3	M4	M5	M6	M7
1	α-Thujene	930	933	0.03	-	-	-	-	-	-
2	α-Pinene	940	940	0.59	1.02	1.33	1.07	1.35	0.46	2.86
3	Camphene	957	955	-	-	-	-	-	0.43	-
4	Sabinene	981	980	0.56	0.79	1.01	0.86	0.99	-	-
5	β-Pinene	985	982	0.93	1.38	6.69	1.16	1.64	0.38	-
6	β-Myrcene	994	993	0.36	0.86	1.33	4.14	1.55	-	-
7	Octan-3-ol	998	996	0.16	0.16	0.42	-	-	-	-
8	α-Phellandrene	1011	1006	-	-	0.71	-	-	-	-
9	α-Terpinene	1023	1024	0.06	-	0.74	0.28	-	-	-
10	*p*-Cymene	1030	1030	0.07	-	2.94	-	0.51	-	-
11	Limonene	1035	1035	-	9.11	1.00	5.64	6.89	0.34	-
12	1,8-Cineole	1039	1038	4.36	8.28	2.66	4.08	6.54	-	5.11
13	(*Z*)-β-Ocymene	1043	1044	0.52	1.25	7.28	0.21	1.58	-	-
14	(*E*)-β-Ocymene	1054	1054	0.12	0.41	1.42	-	0.46	-	-
15	γ-Terpinene	1065	1064	0.12	-	7.59	0.51	-	-	-
16	*cis*-Sabinenehydrate	1074	1074	0.96	0.35	0.11	1.76	-	-	-
17	α-Terpinolene	1092	1097	0.07	-	0.12	-	-	-	-
18	Linalool	1103	1102	0.48	0.11	40.43	-	0.56	-	-
19	Pentyl 3-methylbutanoate	1110	1108	0.06	-	-	-	0.41	-	-
20	Octan-3-yl acetate	1127	1124	-	-	0.27	-	-	-	-
21	Isopulegol	1152	1150	0.1	-	-	-	-	-	-
22	*p*-Menthone	1160	1163	41.00	-	-	-	0.29	-	-
23	Borneol	1172	1172	-	-	-	-	-	1.9	-
24	Menthol	1181	1181	28.19	-	-	-	-	-	-
25	Terpinen-4-ol	1182	1182	0.37	-	0.13	-	-	-	-
26	Isomenthol	1189	1187	0.27	-	-	-	-	-	-
27	Neoisomenthol	1193	1193	0.08	-	-	-	-	-	-
28	α-Terpineol	1194	1194	0.18	0.46	0.93	0.25	-	-	-
29	Myrtenal	1198	1196	0.13	-	-	-	-	-	-
30	*cis*-Dihydrocarvone	1199	1195	-	0.47	-	2.9	26.87	-	-
31	(*Z*)-Hex-3-enyl pentanoate	1240	1236	-	-	0.35	-	-	-	-
32	Pulegone	1244	1244	0.62	-	-	-	-	-	-
33	Carvone	1248	1249	-	62.92	-	72.13	3.95	-	-
34	*cis*-Piperitone oxide	1258	1257	2.82	8.67	0.94	0.28	0.22	21.05	
35	(*E*)-Citral	1274	1271	-	-	0.12	-	-	-	-
36	Menthyl acetate	1278	1278	7.67	-	-	-	-	-	-
37	Neomenthyl acetate	1295	1296	-	-	-	-	0.25	-	-
38	Thymol	1299	1297	0.21	-	7.04	-	0.32	-	-
39	Dihydrocarvyl acetate	1333	1330	-	-	-	0.43	13.01	-	-
40	Piperitenone	1345	1347	-	-	-	-	-	38.93	-
41	Eugenol	1362	1359	0.07	-	-	-	-	-	-
42	*cis*-Carvyl acetate	1367	1365	-	-	-	-	0.23	-	-
43	Piperitenone oxide	1371	1371	-	-	0.51	-	-	32.88	84.66
44	β-Bourbonene	1386	1386	0.13	0.61	0.16	0.87	0.71	-	-
45	β-Elemene	1393	1394	0.07	-	-	0.52	1.58	-	-
46	*cis*-Jasmone	1399	1396	0.06	-	-	-	0.2	0.72	-
47	α-Gurjunene	1411	1411	0.12	-	-	-	-	-	-
48	*trans*-Caryophyllene	1422	1423	0.48	0.92	6.73	0.95	8.21	1.17	2.83
49	α-Humulene	1456	1456	-	-	0.28	-	0.33	-	-
50	(*E*)-β-Farnesene	1460	1460	0.44	-	-	-	-	-	-
51	Germacrene D	1483	1482	2.11	0.72	4.07	0.78	7.35	-	4.54
52	Bicyclogermacrene	1497	1499	0.6	-	0.37	-	-	-	-
53	δ-Cadinene	1526	1524	0.05	-	-	-	-	-	-
54	Elemol	1557	1558	-	-	-	-	5.77	-	-
55	Veridiflorol	1593	1593	0.34	0.31	-	-	2.76	-	-
56	β-Eudesmol	1654	1653	-	-	-	-	0.56	-	-
57	α-Eudesmol	1657	1651	-	-	-	-	0.93	-	-
58	(*E*)-Phytol	2113	2111	0.09	-	-	-	-	-	-

-, Not detected; RI_L_, literature values of retention indices.

**Table 2:** Volatile component composition (peak area%) obtained using HS-SPME method and their amount in *Mentha* spp.

No.	Compound	RI		Sample Symbol and Content (%)						
			RI_L_	M1	M2	M3	M4	M5	M6	M7
1	(*E*)-Hex-2-enal	<900	855	-	0.04	0.16	-	-	-	1.74
2	(*Z*)-Hex-3-en-1-ol	<900	861	-	-	0.21	-	-	-	-
3	α-Thujene	935	933	-	-	0.33	0.08	-	-	-
4	α-Pinene	940	940	0.19	0.25	0.46	0.49	0.33	0.37	0.46
5	Camphene	957	955	-	-	-	-	-	0.37	-
6	Sabinene	981	980	0.23	0.23	0.44	0.55	0.30	0.21	0.44
7	β-Pinene	985	982	0.30	0.36	2.56	0.59	0.43	0.36	3.08
8	Octan-3-one	989	985	-	-	0.09	-	-	-	-
9	β-Myrcene	994	993	0.24	0.44	1.43	3.45	1.03	0.31	-
10	Octan-3-ol	998	996	0.14	0.13	0.59	-	-	0.09	-
11	α-Terpinene	1023	1024	-	-	0.55	0.07	-	-	-
12	*p*-Cymene	1030	1030	0.07	-	3.21	-	0.54	-	-
13	Limonene	1035	1035	2.81	4.74	0.85	5.72	9.34	0.99	-
14	1,8-Cineole	1039	1038	1.94	2.69	1.60	2.23	3.02	0.07	2.75
15	(*Z*)-β-Ocymene	1043	1044	0.55	0.75	6.51	0.17	1.23	-	0.51
16	(*E*)-β-Ocymene	1054	1054	0.13	0.31	1.62	0.06	0.39	-	0.60
17	γ-Terpinene	1065	1064	0.06	-	5.42	0.10	-	-	-
18	*cis*-Sabinenehydrate	1074	1074	0.79	0.11	0.12	2.20	-	-	-
19	*trans*-Linalool oxide	1078	1080	-	-	0.15	-	-	-	-
20	α-Terpinolene	1092	1097	0.05	-	-	0.06	-	0.04	-
21	*trans*-Sabinene hydrate	1102	1101	-	-	-	0.09	-	-	-
22	Linalool	1103	1102	0.23	-	45.24	-	0.76	-	0.38
23	Pentyl 3-methylbutanoate	1110	1108	0.07	-	-	-	0.38	-	-
24	Octan-3-yl acetate	1127	1124	0.08	-	0.48	-	-	-	0.60
25	Alloocimene	1135	1131	-	-	0.12	-	-	-	-
26	*p*-Mentha-1,5,8-triene	1136	1135	-	-	0.17	-	0.23	-	-
27	*trans*-Limonene oxide	1143	1141	-	-	-	0.07	-	-	-
28	Isopulegol	1152	1150	0.07	-	0.18	-	-	-	-
29	Citronellal	1158	1158	-	-	5.32	-	-	-	-
30	*p*-Menthone	1160	1163	27.64	-	-	-	0.39	-	-
31	Menthofuran	1170	1169	7.27	-	-	-	-	-	-
32	Neomenthol	1171	1167	3.67	-	-	-	-	-	-
33	Borneol	1172	1172	-	-	-	-	-	1.56	-
34	*δ*-Terpineol	1173	1171	-	0.15	-	0.15	-	-	-
35	Menthol	1181	1181	26.48	-	-	-	-	-	-
36	Terpinen-4-ol	1182	1182		-	-	0.05	-	-	-
37	Isomenthol	1189	1187	0.40	-	-	-	-	-	-
38	Neoisomenthol	1193	1193	0.12	-	-	-	-	-	-
39	α-Terpineol	1194	1194	-	0.37	1.06	-	-	-	-
40	Myrtenal	1198	1196	0.06	-	-	-	-	0.18	-
41	*cis*-Dihydrocarvone	1199	1195	-	0.55	-	1.39	39.11	-	-
42	*trans*-Dihydrocarvone	1207	1206	-	-	-	0.09	2.51	-	-
43	*trans*-Carveol	1227	1230	-	-	-	0.13	-	-	-
44	β-Cytronelol	1233	1232	-	-	0.19	-	-	-	-
45	(*Z*)-Hex-3-enyl pentanoate	1240	1236	-	-	0.34	0.26	-	-	0.46
46	(*Z*)-Citral	1243	1245	-	-	1.06	-	-	-	-
47	Pulegone	1244	1244	3.67	-	-	-	-	-	-
48	Carvone	1248	1249	0.08	74.49	0.16	71.44	11.98	-	0.52
49	*cis*-Piperitone oxide	1258	1257	3.51	10.17	1.21	0.40	0.48	23.21	-
50	(*E*)-Citral	1274	1271	-	-	1.93	-	-	-	-
51	Menthyl acetate	1278	1278	0.68	-	-	-	-	-	-
52	Neomenthyl acetate	1295	1296	14.13	-	-	-	-	-	-
53	Thymol	1299	1297	0.16	-	5.48	-	-	0.20	-
54	Neoisomenthyl acetate	1310	1311	0.15	-	-	-	-	-	-
55	Dihydrocarvyl acetate	1333	1330	-	-	-	0.15	10.70	-	-
56	*trans*-Carvyl acetate	1342	1342	-	-	-	0.09	-	-	-
57	Piperitenone	1345	1347	-	-	-	-	-	34.68	-
58	*cis*-Carvyl acetate	1367	1365	-	-	-	0.15	0.19	-	-
59	Piperitenone oxide	1371	1371	-	-	1.04	-	-	30.96	72.69
60	α-Copaene	1379	1378	0.13	-	-	-	-	-	-
61	β-Bourbonene	1386	1386	0.15	0.88	0.08	1.21	0.52	-	-
62	β-Elemene	1393	1394	0.11	-	-	0.89	0.84	-	2.22
63	*cis*-Jasmone	1399	1396	-	-	-	0.26	-	0.78	
64	Tetradecane	1400	1400	0.10	0.39	0.31	-	0.66	-	1.63
65	α-Gurjunene	1411	1411	0.10	-	-	-	-	-	-
66	*trans*-Caryophyllene	1422	1423	0.39	0.90	5.15	1.73	6.00	1.49	2.63
67	α-Humulene	1456	1456	-	-	0.20	0.06	0.21	0.10	-
68	(*E*)-β-Farnesene	1460	1460	0.52	0.22	0.12	0.37	-	0.52	0.93
69	α-Amorphene	1479	1485	0.05	-	-	0.06	0.32	0.41	-
70	Germacrene D	1483	1482	1.24	0.34	1.36	1.56	3.11	-	1.54
71	Bicyclogermacrene	1497	1499	0.47	-	0.19	0.44	-	-	-
72	*δ*-Cadinene	1526	1524	0.12	-	0.28	0.18	0.55	-	-
73	Elemol	1557	1558	-	-	-	-	0.51	-	-
74	Veridiflorol	1593	1593	0.11	-	-	-	0.32	-	-
75	α-Eudesmol	1657	1651	-	-	-	-	0.35	-	-

-, Not detected; RI_L_, literature values of retention indices.

## Data Availability

All data reported here are available from the authors upon request.

## Sample Availability

Samples of the compounds are not available from the authors.
